# Reduction of Defects in AlGaN Grown on Nanoscale-Patterned Sapphire Substrates by Hydride Vapor Phase Epitaxy

**DOI:** 10.3390/ma10060605

**Published:** 2017-05-31

**Authors:** Chi-Tsung Tasi, Wei-Kai Wang, Tsung-Yen Tsai, Shih-Yung Huang, Ray-Hua Horng, Dong-Sing Wuu

**Affiliations:** 1 Department of Materials Science and Engineering, National Chung Hsing University, Taichung 40227, Taiwan; d100066018@mail.nchu.edu.tw (C.-T.T.); d9566208@alumni.nchu.edu.tw (T.-Y.T.); 2 Department of Materials Science and Engineering, Da-Yeh University, Changhua 51591, Taiwan; wk@mail.dyu.edu.tw; 3 Department of Industrial Engineering and Management, Da-Yeh University, Changhua 51591, Taiwan; syh@mail.dyu.edu.tw; 4 Department of Electronics Engineering, National Chiao Tung University, Hsinchu 300, Taiwan; rhh@nctu.edu.tw

**Keywords:** AlGaN, hydride vapor phase epitaxy (HVPE), nanoscale-patterned sapphire substrate (NPSS), dislocation density (TD)

## Abstract

In this study, a 3-μm-thick AlGaN film with an Al mole fraction of 10% was grown on a nanoscale-patterned sapphire substrate (NPSS) using hydride vapor phase epitaxy (HVPE). The growth mechanism, crystallization, and surface morphology of the epilayers were examined using X-ray diffraction, transmission electron microscopy (TEM), and scanning electron microscopy at various times in the growth process. The screw threading dislocation (TD) density of AlGaN-on-NPSS can improve to 1–2 × 10^9^ cm^−2^, which is significantly lower than that of the sample grown on a conventional planar sapphire substrate (7 × 10^9^ cm^−2^). TEM analysis indicated that these TDs do not subsequently propagate to the surface of the overgrown AlGaN layer, but bend or change directions in the region above the voids within the side faces of the patterned substrates, possibly because of the internal stress-relaxed morphologies of the AlGaN film. Hence, the laterally overgrown AlGaN films were obtained by HVPE, which can serve as a template for the growth of ultraviolet III-nitride optoelectronic devices.

## 1. Introduction

Recently, the use of epitaxial Al*_x_*Ga_1 −* x*_N alloys wide-band gap semiconductors have drawn increasing attention because of their potential expanding applications for ultraviolet (UV) optoelectronic devices and high-power, high-frequency electronic devices [1,2,3]. However, the dislocation density within UV optoelectronic devices significantly affects their internal quantum efficiency and operational lifetime due to the presence of heteroepitaxial-growth-induced defects (e.g., threading dislocations (TDs), stacking faults, voids, and point defects) [4,5]. Hence, the epitaxial growth of high quality AlGaN materials plays an important role in determining the performance of AlGaN-based devices. 

Hydride vapor phase epitaxy (HVPE) has been shown to achieve rapid growth rates of several hundred micrometers per hour. Growth rates of approximately 30–50 µm/h have been reported for the growth of freestanding GaN substrates and AlN [6,7]. Therefore, HVPE is expected to permit the growth of thick AlGaN, which can possibly serve as pseudosubstrates to mitigate the lack of suitable substrates for the growth of low-defect AlGaN wafer. However, AlGaN pseudosubstrates are not currently available because of practical fabrication challenges, including crack formation within the Al*_x_*Ga_1−*x*_N layer grown on foreign substrates, inhomogeneous Al incorporation at different facets, and parasitic reactions [8]. Al-containing material tends to react violently with SiO_2_ during growth, and thus a SiO_2_ mask is forbidden to use for AlGaN epitaxial lateral overgrowth (ELOG). To address these issues, patterned sapphire substrates (PSSs) are promising substrate materials for the direct lateral epitaxial growth of AlGaN using HVPE [9,10]. Hedagorn et al. reported the successful growth of a smooth 40-μm-thick Al_0.45_Ga_0.55_N layer on a trench-shaped PSS. They also investigated the effect of the total pressure, V/III ratio, and substrate miscut direction on the growth of AlGaN on a trench-shaped PSS [11,12]. Kuwano et al. showed that the main problem in ELOG is predominantly caused by the grain growth along different orientations on the sidewalls of the patterned substrates [13]. Richter et al. discussed the growth behavior of an Al_0.3_Ga_0.7_N layer grown on a honeycomb-shaped PSS [14]. They observed that nanometer-scaled patterns rather than micrometer-scale stripe patterns are more suitable for the extraction of UV emitter light through the substrate. Furthermore, patterned structures extending to the nanoscale (submicron) are more effective in reducing the number of TDs by bending the dislocation direction to the sidewalls of the patterned template [15]. In this study, an overgrown high-quality AlGaN layer was directly grown on nanoscale-PSSs (NPSSs) using HVPE without the use of complex processes. In addition, the growth mechanism and material properties of the AlGaN were investigated as a function of growth time.

## 2. Materials and Methods 

Two-inch c-plane sapphire substrates were used as the starting materials for the NPSSs. A low-pressure chemical vapor-deposited SiO_2_ film on sapphire served as the mask layer, which the nano-imprint resist was spin-coated on. The hexagonal hole array was transferred to the resist by nano-imprint lithography, followed by oxygen plasma descum to removing any residual resist at the bottom of the holes. The SiO_2_ film was then etched by a fluorine plasma. Finally, a BCl_3_/Cl_2_ high-density-plasma etching process was employed to etch the sapphire substrate, and the mask was removed by a dilute HF solution. Although a variety of hole dimensions for nano-imprinting were attempted, the optimum NPSSs used in this study were with 500-nm-diameter hole array, spaced 950 nm apart, and etched to a depth of 400 nm. 

**Figure 1:**
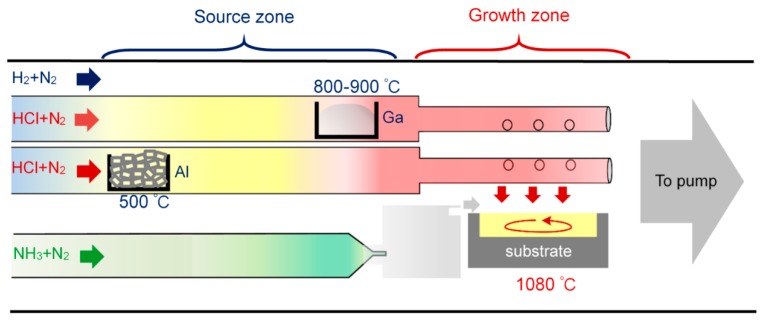
Schematic diagram of the hydride vapor phase epitaxy (HVPE) reactor used for the AlGaN growth.

The growth of the AlGaN epilayer on NPSS or conventional sapphire substrate (CSS) was performed using a HVPE horizontal reactor as shown schematically in Figure 1. The quartz glass reactor was covered with a furnace containing five heating zones maintained at different temperatures. Ga and Al metal chlorides serving as the group III Ga and Al precursor sources, respectively, were separately placed in the upstream region of the quartz reactor. The AlCl_3_ and GaCl vapors were generated in the reactor by flowing HCl over the Al (10 sccm) and Ga precursor (10 sccm) sources, respectively. To avoid the formation of AlCl vapor by a reaction between the Al metals and HCl at high temperature (which would damage the quartz reactor), the Al metal source was maintained at 500 °C to ensure the sole formation of AlCl_3_ vapor. The temperature of the GaCl source was maintained between 800 °C and 900 °C. Pure N_2_ gas (400 sccm) served as the carrier gas to propel the AlCl_3_ and GaCl vapors through two quartz tubes to the growth zone. The ammonia line consists of the NH_3_ flow (2 L/min) and N_2_ flow (300 sccm). During the HVPE process, the H_2_ flow was kept at 2.45 L/min, N_2_ flow at 200 sccm, growth pressure at 200 mbar, and growth temperature at 1080 °C. Transmission electron microcopy (TEM, JEM-2010, JEOL, Tokyo, Japan), scanning electron microscopy (SEM, S-3000H, Hitachi, Tokyo, Japan), atomic force microscopy (AFM, 5400, Agilent, Santa Clara, CA, USA), double-crystal x-ray diffraction (DCXRD, X’Pert PRO MRD, PANalytical, Almelo, The Netherlands), and photoluminescence (PL, Flouromax-3, Horiba, Tokyo, Japan) measurements were conducted to examine the microstructure and optical properties of the AlGaN epilayers grown on the NPSSs.

## 3. Results and Discussion

**Figure 2:**
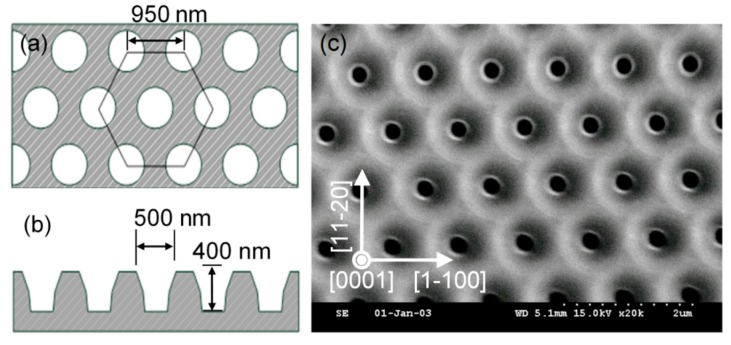
Schematic illustrations of the (**a**) top- and (**b**) side-view dimensions of the nanoscale- patterned sapphire substrate (NPSS) design used in this study. (**c**) SEM surface morphology of the as-etched NPSS.

Schematic illustrations of the top- and side-view dimensions of the NPSS design are shown in Figure 2a,b, respectively. A typical surface morphology of the as-etched NPSS examined by scanning electron microscopy (SEM) is shown in Figure 2c. To study the patterned substrate effect, the HVPE growth of the Al*_x_*Ga_1−*x*_N with an Al mole fraction of *x* = 0.1 (“AlGaN” hereafter) on NPSS was proceeded. A CSS was also used in these experiments.

**Figure 3:**
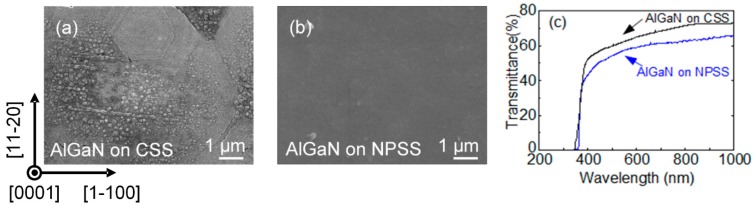
SEM surface morphologies of the AlGaN epilayers grown on the (**a**) conventional sapphire substrate (CSS) and (**b**) NPSS. The corresponding transmittance spectra are shown in (**c**).

The SEM surface morphologies of the AlGaN films grown on both the CSS and NPSS are shown in Figure 3a,b, respectively. The AlGaN on CSS exhibited a hexagonal-shape faceting structure, resulting in a rough surface. In contrast, the AlGaN film grown on an NPSS shows a relatively smooth and uniform surface. A comparison of the transmittance spectra of AlGaN grown on both the CSS and NPSS is shown in Figure 3c. The optical absorption edge at 360 ± 5 nm corresponds to an Al mole fraction of 10 at %, which is consistent with our double-crystal X-ray diffraction (DCXRD) measurements. In general, the AlGaN layer on the NPSS exhibited reduced transmissivity, which is thought to be caused by light scattering via the NPSS and reflections caused by air voids embedded at the interface between the AlGaN and sapphire interface [10].

**Figure 4:**
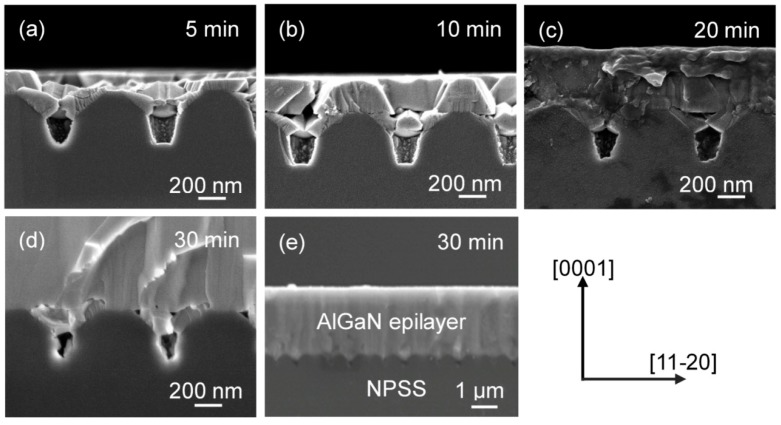
Cross-sectional SEM images of the AlGaN epilayers on NPSSs for various growth times ((**a**–**d**) 5, 10, 20, and 30 min); (**e**) are micrographs of the same 3-μm-thick cross-sectional AlGaN-on-NPSS sample (30 min) with different magnifications.

To study the nanoheteroepitaxial growth of AlGaN-on-NPSS, the growth evolution with various growth periods were identified by cross-sectional SEM images. Figure 4a–d show the AlGaN films grown on NPSSs at growth times of 5, 10, 20, and 30 min. For the initial growth times of 5 and 10 min, the AlGaN films began to grow from the NPSS sidewall of m-plane facets (Figure 4a–b). The selective growth of the AlGaN layer is possibly related to the different migration rates of Ga atoms on the (0001) and {11−2*k*} facets during the HVPE growth process. With time, the AlGaN coalesced, resulting in the formation of an epitaxial lateral overgrowth (Figure 4c). Finally, after 30 min, a flat 3-μm-thick AlGaN epilayer on a NPSS with small voids at the interface is shown in Figure 4d–e with different magnifications.

**Figure 5:**
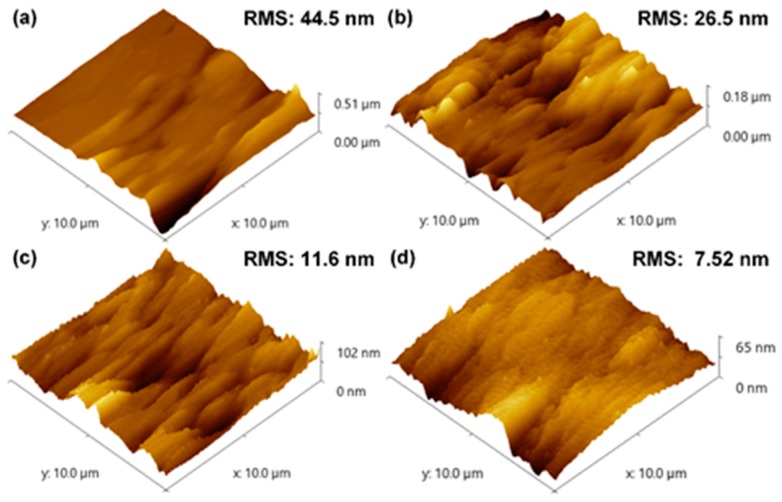
Atomic force microscopy (AFM) measurement of AlGaN grown on an NPSS at growth times of (**a**) 5; (**b**) 10; (**c**) 20 and (**d**) 30 min. RMS: root-mean-square.

The corresponding surface roughnesses of these samples were examined by atomic force microscopy using a scan area of 100 μm^2^. As shown in Figure 5, the surface root-mean-square (RMS) roughness values of these samples were measured to be 44.5, 26.5, 11.6, and 7.52 nm for growth times of 5, 10, 20, and 30 min, respectively. The AFM results indicate that the surface of the AlGaN film on the NPSS was relatively smooth and did not contain surface pits at a growth time of 30 min. 

**Figure 6:**
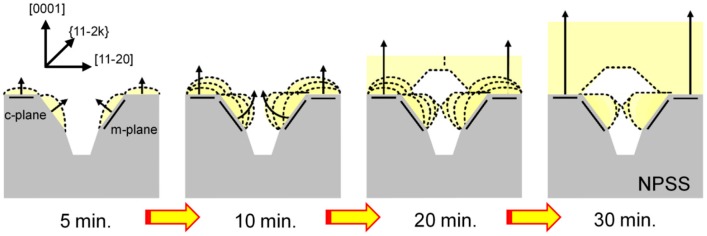
AFM measurement of AlGaN grown on a NPSS at growth times of 5, 10, 20, and 30 min.

Based on these observations, a schematic growth evolution mechanism of the AlGaN ELOG structure on the NPSS is proposed and shown in Figure 6. The large overgrowth formation would lead to a reduction in the TD density in the overlayer region. However, the AlGaN film formed at an initial growth time of 5 min exhibited a very rough surface, indicating an incomplete 3D-island-coalescence growth process. This result is consistent with the SEM results presented in Figure 4a.

**Figure 7:**
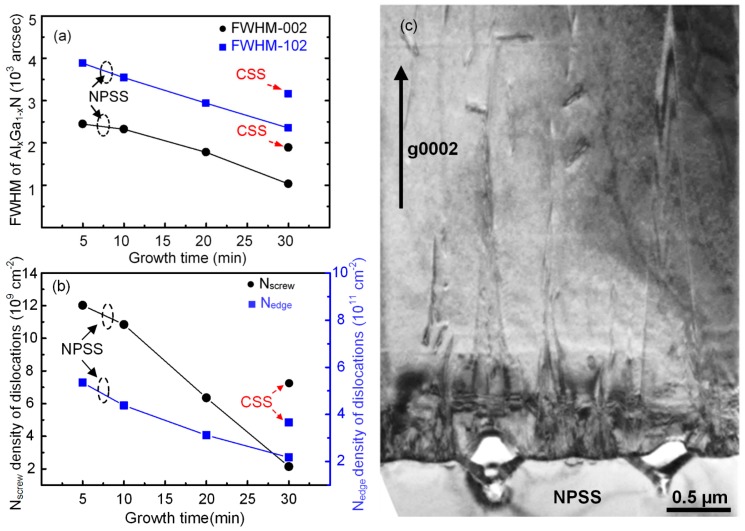
(**a**) Double-crystal x-ray diffraction (DCXRD) full-widths at half-maximum (FWHMs) of (0002)- and (10-12)-AlGaN on NPSSs for various growth times (5, 10, 20, and 30 min); (**b**) Calculated screw and edge dislocation densities of AlGaN samples on NPSSs for various growth times. The data for HVPE AlGaN sample grown on a CSS for 30 min are also depicted for comparison; (**c**) Bright field TEM image of 3-μm-thick AlGaN epilayer grown on an NPSS with g = 0002. The corresponding threading dislocation densities in various depths are also measured.

Figure 7a shows the DCXRD full-width half-maximum (FWHM) results with the symmetric (002) and asymmetric (102) planes for the AlGaN samples as demonstrated in Figure 4. The FWHM values of the (002) and (102) reflections decreased with increasing growth time from 5 to 30 min. At a growth time of 30 min, narrower FWHM values of 1000 and 2500 arcsec were obtained for the (002) and (102) reflections, respectively, indicating that the quality of the AlGaN epilayer improved for a lower TD density. Furthermore, the symmetric (002) and (102) reflections can provide information on the density of pure screw and pure edge TDs, respectively. The reflection was brought into the diffraction condition by rotating the crystal’s surface normal out the diffraction plane by an angle 𝜒. Here the angle 𝜒 was measured at 48.08 between the reciprocal lattice vector (K_hkl_, hkl = 102) and (001) surface normal. Specifically, the density of screw dislocations *ρs* could be calculated using the following equation:

(1) $$\rho s\;=\frac{\beta_{\mathrm{tilt}}^{2}}{4.35b_{c}^{2}}$$

where *b_c_* is the Burgers vector of c-type TDs (*b_c_* = 0.5185 nm) and the FWHM values of (002) reflection were used to evaluate the tilt angle *β*_tilt_ from the XRD rocking curve [16]. The twist angle *β*_twist_ was estimated according to the method outlined previously by Lee et al. [17].

(2) $$\beta =\sqrt{{\left(\beta_{\mathrm{tilt}}cos\chi \right)}^{2}+{\left(\beta_{\mathrm{twist}}Sin\chi \right)}^{2}}$$

where the values of *β* were calculated for the FWHM values of the (102) reflection. The twist angle *β*_twist_ was calculated from XRD data according to Equation (2). The density of edge dislocations *ρe* was estimated using the following equation [16]:

(3) $$\rho e\;=\;\frac{\beta_{\mathrm{twist}}^{2}}{4.35b_{a}^{2}}$$

where *b_a_* is the Burgers vector of a-type TDs (*b_a_* = 0.1503 nm). Moreover, the Burgers vector of mixed dislocations (a + c)-types (*b_m_*) can be composed into pure screw and edge components: *b_m_* = *b_c_* + *b_a_*. In this study, these dislocations are not considered separately [18]. Figure 7b shows the corresponding dislocation density determined from the DCXRD (002) and (102) reflections. At a growth time of 30 min, the AlGaN film on the NPSS exhibited a lower screw dislocation density of 2 × 10^9^ cm^–2^ compared with that on the CSS (7 × 10^9^ cm^–2^). Better crystallinity and lower TD might be attributed to the strain relaxation and dislocation reduction in the AlGaN/NPSS interface. To investigate the crystalline quality and local defect distribution of the AlGaN layer grown on the NPSS, TEM analysis was performed. Figure 7c presents a bright-field cross-sectional TEM image of the AlGaN layer grown on the NPSS under a two beam condition with g = 0002. The pure screw and mixed screw/edge defects are visible under the g = 0002 two beam condition [19]. From the unpatterned substrate region, a large number of typical extended TDs propagated throughout the AlGaN film, originating at the interface between the AlGaN and sapphire. These TDs were generated by the large lattice mismatch between the AlGaN and sapphire. However, the voids and TDs bending observed within the NPSS indicate freestanding overgrowth in the lateral direction. Finally, TDs in the region above these voids were seldom observed. The results of TEM analysis indicate that the AlGaN growth on the NPSS effectively reduced TDs in the epitaxial layer. This observation is similar to that reported in previous studies of PSS/ELOG [20].

**Figure 8:**
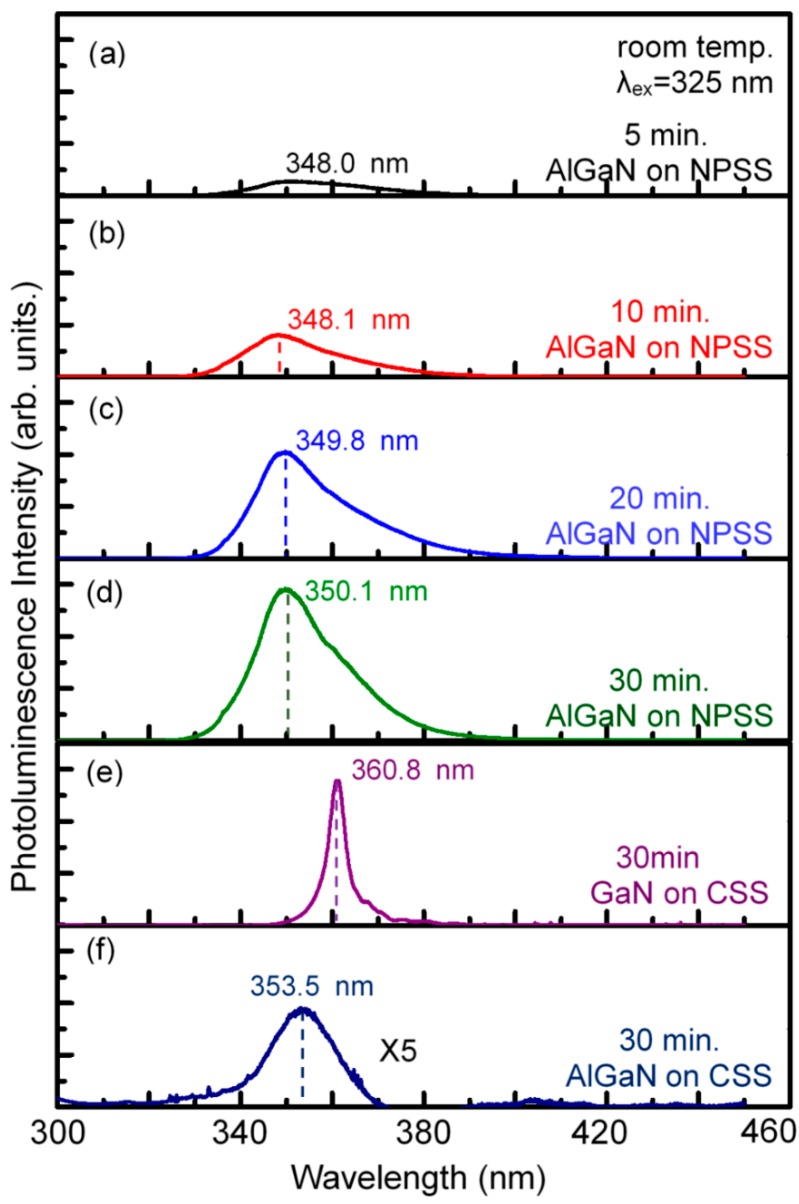
Room-temperature photoluminescence spectra of the HVPE AlGaN-on-NPSS epilayers as a function of growth time (**a**) 5; (**b**) 10; (**c**) 20 and (**d**) 30 min. The HVPE GaN and AlGaN samples on CSS for 30 min are also presented in (**e**,**f**) for comparison.

A prime concern about the AlGaN heteroepilayers is their optical properties revealed by photoluminescence (PL) measurements. Figure 8a–d show the room-temperature PL emission spectra of AlGaN films grown on NPSSs for various growth times (5–30 min). For comparison, the PL spectra of the 3-μm-thick GaN and AlGaN samples on CSSs by HVPE are also presented in Figure 8e,f, respectively. The intense peak corresponding to near-band-edge emission at approximately 350 nm is related to the growth of AlGaN-on-NPSS sample, whereas 360.8 nm is related to GaN-on-CSS and 353.5 nm is related to AlGaN-on-CSS samples. As the growth time increased from 10 to 30 min, a redshift from 348 to 350 nm in the PL emission peak position was observed, corresponding to the grain-size confinement effect [21]. The redshift of the PL emission from 350 to 353.5 nm could be due to stress relaxation, where the AlGaN-on-CSS sample with a higher TD density has been confirmed as compared with the AlGaN-on-NPSS one (Figure 7b). Moreover, the PL peak intensity increased with increasing growth time. The increase in the PL peak intensity accompanied by the decrease in the number of defects indicates an improvement of the crystalline quality of the AlGaN epilayer and a reduction of the defect density. The PL measurements indicate that the crystallinity and optical quality of the AlGaN films grown on NPSSs improved with increasing growth time.

## 4. Conclusions

In this study, the growth mechanism and crystal quality of AlGaN epilayers grown on NPSSs were investigated. SEM images revealed the dominant growth of (11−22) plane AlGaN from m-plane sidewalls of the NPSSs, and optical transmittance spectrum measurements showed that the AlGaN epilayer exhibited UV transparency for wavelengths above 360 nm. TEM analysis revealed that the annihilation of TDs was related to the formation of stacking faults resulting from the different strains present at the grain boundary between the AlGaN and NPSS. These results demonstrate the high potential of HVPE AlGaN/NPSS as the epitaxial template for fabricating high-performance AlGaN-based optoelectronics devices.
